# Inhibitors of Protein Glycosylation Are Active against the Coronavirus Severe Acute Respiratory Syndrome Coronavirus SARS-CoV-2

**DOI:** 10.3390/v13050808

**Published:** 2021-04-30

**Authors:** Sreejith Rajasekharan, Rafaela Milan Bonotto, Lais Nascimento Alves, Yvette Kazungu, Monica Poggianella, Pamela Martinez-Orellana, Natasa Skoko, Sulena Polez, Alessandro Marcello

**Affiliations:** 1 Laboratory of Molecular Virology, International Centre for Genetic Engineering and Biotechnology (ICGEB) Padriciano, 99-34149 Trieste, Italy or rajasekharan@leibniz-hpi.de (S.R.); Rafaela.Bonotto@icgeb.org (R.M.B.); Lais.Nascimento@icgeb.org (L.N.A.); kazungu@icgeb.org (Y.K.); poggiane@icgeb.org (M.P.); Pamela.Martinez@icgeb.org (P.M.-O.); 2 Biotechnology Development, International Centre for Genetic Engineering and Biotechnology (ICGEB) Padriciano, 99-34149 Trieste, Italy; skoko@icgeb.org (N.S.); polez@icgeb.org (S.P.)

**Keywords:** COVID-19, SARS-CoV-2, coronavirus, inhibitor, antiviral, Miglustat, spike, Celgosivir

## Abstract

Repurposing clinically available drugs to treat the new coronavirus disease 2019 (COVID-19) is an urgent need in the course of the Severe Acute Respiratory Syndrome coronavirus (SARS-CoV-2) pandemic, as very few treatment options are available. The iminosugar Miglustat is a well-characterized drug for the treatment of rare genetic lysosome storage diseases, such as Gaucher and Niemann-Pick type C, and has also been described to be active against a variety of enveloped viruses. The activity of Miglustat is here demonstrated in the micromolar range for SARS-CoV-2 in vitro. The drug acts at the post-entry level and leads to a marked decrease of viral proteins and release of infectious viruses. The mechanism resides in the inhibitory activity toward α-glucosidases that are involved in the early stages of glycoprotein N-linked oligosaccharide processing in the endoplasmic reticulum, leading to a marked decrease of the viral Spike protein. Indeed, the antiviral potential of protein glycosylation inhibitors against SARS-CoV-2 is further highlighted by the low-micromolar activity of the investigational drug Celgosivir. These data point to a relevant role of this approach for the treatment of COVID-19.

## 1. Introduction

The novel Severe Acute Respiratory Syndrome coronavirus (SARS-CoV-2), the etiologic agent of coronavirus disease 2019 (COVID-19), has now spread worldwide causing a global pandemic [1,2]. To date there have been more than 120 million confirmed cases and almost 3 million deaths worldwide spurring a global effort to tackle the disease [3]. SARS-CoV-2 belongs to the genus Betacoronavirus of the order/family/sub-family Nidovirales/Coronaviridae/Coronavirinae [4]. The virion is enveloped and contains a single RNA genome of positive polarity. 

Morphologically, SARS-CoV-2 is about 120 nm in diameter with large projections of heavily glycosylated trimeric Spike (S) proteins. Other surface proteins include the membrane (M) and envelope (E) proteins, while, inside the envelope, the helical nucleocapsid (N) wraps the viral RNA.

The virus targets cells of the upper and lower respiratory tract epithelia through the viral Spike that binds to the angiotensin-converting enzyme 2 (ACE2) receptor, a process facilitated by the host type 2 transmembrane serine protease, TMPRSS2. Once inside the cell, viral polyproteins are synthesized that encode for the replication machinery required to synthesize new RNA via the RNA-dependent RNA polymerase activity. 

Replication is cytoplasmatic at the level of the endoplasmic reticulum (ER), which is heavily rearranged. Structural proteins are then synthesized leading to the completion of assembly and the release of viral particles [5,6]. Currently, specific antiviral treatment against SARS-CoV-2 is limited to the repurposed drug remdesivir, which received emergency use authorization for COVID-19 treatment; however, debate on its real efficacy remains open. Indeed, in addition to remdesivir, several antiviral drugs being proposed are from the repurposing of drugs developed for other viral infections. 

Lopinavir, ritonavir, (hydroxy)chloroquine, umifenovir, and favipiravir are examples that are currently being evaluated; however, none have been conclusively shown to be effective [7]. A recent addition to the armamentarium of strategies to inhibit SARS-CoV-2 is represented by neutralizing monoclonal antibodies, although their narrow window of applicability and sensitivity to Spike immunological escape mutations makes them non-resolutive [8].

The iminosugar Miglustat (Zavesca; *N*-butyl-1-deoxynojirimycin, NB-DNJ) inhibits α-glucosidases I and II, which are involved in the early stages of glycoprotein N-linked oligosaccharide processing in the ER [9]. As most enveloped viruses require glycosylation for surface protein folding and secretion, modulation of the oligosaccharides to induce a reduction in infectivity is a strategy for the treatment of immune deficiency virus type 1 (HIV-1), culminating in phase I/II clinical trials [10,11]. 

The use of iminosugars to misfold viral glycoprotein as a therapeutic approach has thus far been applied to several other viral infections including: hepatitis B and C viruses, Dengue and other flaviviruses, and Ebola virus [12,13,14]. An additional property of certain iminosugars is the glucosyltransferase inhibition activity, which is the basis for the current therapy of rare genetic lysosome storage diseases, such as Gaucher and Niemann-Pick type C [15]. This activity of Miglustat could impact virus entry by modification of the plasma membrane.

Celgosivir is an investigational prodrug of the natural α-glucosidases I inhibitor castanospermine, which was initially developed, similarly to Miglustat, as an HIV-1 inhibitor up to Phase I–II clinical trials [16,17]. Phase II clinical trials have also been conducted in patients with hepatitis C virus infection and showed poor efficacy as monotherapy but were synergistic in combination with pegylated Interferon α-2b [18]. With the advent of highly effective direct acting antivirals for HCV, the use of interferon and associated drugs is not the primary recommended treatment option for HCV. 

Finally, the Phase 1b CELADEN trial investigated the safety and efficacy of Celgosivir in patients with Dengue fever [19]. Although safe and well tolerated, Celgosivir did not significantly reduce the viral load or fever burden in Dengue patients. However, early intervention may be required as shown in animal studies [20]. Careful evaluation of the CELADEN trial suggested that new dosing regimens could achieve better responses in patients with secondary Dengue infection [21]. 

The wealth of data on the clinical use of Miglustat for the treatment of lysosomal storage disorders and the antiviral properties observed on enveloped viruses make Miglustat an ideal candidate of drug repurposing for COVID-19. In this work, Miglustat was shown to be active for the inhibition of SARS-CoV-2 in different cell types at concentrations compatible with those obtained for the treatment of Gaucher and Niemann-Pick type C in patients. 

Time of addition studies indicated that the inhibitory activity was at the post-entry level and affected the release of infectious viruses. The proper folding and release of the Spike protein and progeny virus appeared to be affected. In addition, the glycosylation inhibitor Celgosivir, which has also been shown to be active against various viruses, has been tested for SARS-CoV-2 and showed potent activity. These data further highlight the opportunity of using inhibitors of this essential pathway for the treatment of COVID-19.

## 2. Materials and Methods

### 2.1. Cells, Virus and Antiviral Assay

Vero E6 cells (ATCC-1586) HEK 293T (ATCC CRL-3216), A549 (ATCC CCL-185), U2OS (ATCC HTB-96), human hepatocarcinoma Huh-7 cells kindly provided by Ralf Bartenschlager (University of Heidelberg, Heidelberg, Germany), lung adenocarcinoma Calu-3 (ATCC HTB-55), and Huh-7 cells engineered by lentivirus transduction to overexpress the human ACE2 (Huh7-hACE2) [22] were cultured in Dulbecco’s modified Eagle’s medium (DMEM, ThermoFisher, Paisley, UK ) supplemented with 10% foetal bovine serum (FBS, ThermoFisher, Paisley, UK) and antibiotics. Cell cultures were maintained at 37 °C under 5% CO2. Cells were routinely tested for mycoplasma contamination.

Working stocks of SARS-CoV-2 ICGEB-FVG_5 isolated in Trieste, Italy, were routinely propagated and titrated on Vero E6 cells [23]. Plaque assay was performed by incubating dilutions of SARS-CoV-2 on Vero E6 monolayers at 37 °C for 1 h, which were then washed with phosphate buffered saline (PBS) and overlaid with DMEM 2% FBS containing 1.5% carboxymethylcellulose (CMC, Sigma-Aldrich, St Louis, USA) for 3 days. The cells were then fixed with 3.7% paraformaldehyde (PFA, Sigma-Aldrich, St Louis, MO, USA) and stained with crystal violet 1%. A cytotoxicity assay was performed with Alamar Blue (ThermoFisher, Eugene, OR, USA) according to the manufacturer’s instructions. 

### 2.2. Drugs and Proteins

Miglustat (NB-DNJ) and Celgosivir (MX-3253, MBI-3253) were purchased from Sigma-Aldrich, St Louis, MO, USA (B8299 and SML2314, respectively). Miglustat was dissolved in DMSO to obtain a stock solution, while Celgosivir was dissolved in distilled water. 

The SARS-CoV-2 Spike protein receptor-binding domain (RBD) was expressed from pCAGGS using a construct generously provided by Florian Krammer (Mount Sinai, New York, NY, USA) [24]. The plasmid was transfected in 293T cells, and cell extracts and supernatants were harvested at 24 h post-transfection. Miglustat 200 μM was added after transfection and maintained in the medium until the end of the experiment.

Sequence coding for the full-length Spike protein was obtained from the isolate Wuhan-Hu-1 (NCBI Reference Sequence: NC_045512.2). The nucleotide sequence, fused to an immunoglobulin leader sequence (sec) at the N-terminus, with the codon optimized for expression in mammalian cells, was obtained as a synthetic DNA fragment from GenScript Biotech (Leiden, The Netherlands) and cloned as HindIII/ApaI into a pCDNA3 vector.

### 2.3. Plaque Reduction Assay

Vero E6 cells were seeded at 6 × 10^4^ cells/well density in a 48-well plate and incubated at 37 °C overnight. The cells were infected with 30 viral PFU/well and incubated at 37 °C for 1 h. Following incubation, the virus was removed, and the wells washed with 1× PBS. The infected cells were maintained with 800 μL of overlay medium containing 1.5% CMC with DMEM + 2% heat-inactivated FBS, and Miglustat dilutions. The cells were then incubated at 37 °C for 3 days. Finally, the cells were fixed with 3.7% PFA and stained with crystal violet. 

The plaques were counted, and the values were normalized to the vehicle (DMSO). The plaque reduction assays were conducted in double replicates for three independent experiments. The inhibition was calculated with the formula: (1-(average plaques Miglustat/average plaques Vehicle)) × 100 and plotted against dilutions as the antilog. For the cytotoxicity assay, the fluorescence readings were normalized for vehicle and percent plotted against the dilutions. The half maximal effective concentration (EC_50_) and cytotoxic concentration (CC_50_) were calculated using GraphPad Prism Version 7.

### 2.4. Immunofluorescence, Immunoblotting, and Flow-Cytometry

A recombinant monoclonal reactive with the receptor-binding domain of the S protein was generated based on a mouse small immune protein (SIP) scaffold (mSIP-3022) [25]. The DNA fragment encoding for the variable regions VL (NCBI accession code: DQ168570.1) and VH (NCBI accession code: DQ168569.1) of human monoclonal antibodies, clone CR3022 [26,27], was synthetized as scFV by GenScript Biotech (Leiden, Netherlands) and cloned into ApaLI-BspEI sites upstream of the Hinge-CH2-CH3 domains of the mouse IgG2b expression vector as described previously [28]. 

The plasmid was transfected in ExpiCHO-S cells (Life-Technologies, Bleiswijr, The Netherlands) following the manufacturer’s instructions. Eight days post transfection, the supernatant was loaded on the HiTrap protein G HP 1 mL column (GE Healthcare, Chicago, IL, USA) in binding buffer (20 mM sodium phosphate pH 7.0) and eluted with acetic acid 50 mM pH 2.7. The eluted antibody was immediately neutralized with 1 M Tris pH 8, and analysed by RP-HPLC and by SDS PAGE to maintain the dimeric structure. The production yield was 0.8 mg/mL. For immunofluorescence, the cells were fixed in 3.7% PFA, permeabilized with 0.1% Triton and processed with mSIP-3022 as per standard procedure [29]. 

Since mSIP-3022 did not react with the denatured S protein, a convalescent serum from a COVID-19 patient was used for immunoblotting at a 1:200 dilution. The images were acquired on a Zeiss LSM880 confocal microscope. For immunoblotting, whole-cell lysates were resolved by 12% SDS-PAGE and blotted onto nitrocellulose membranes. The membranes were blocked in 5% nonfat milk in Tris-buffered saline (TBS) plus 0.1% Tween 20 (TBST), followed by incubation with the human serum diluted 1:200 in the same solution for 1 h at room temperature. 

After washing three times with TBST, secondary horseradish peroxidase (HRP)-conjugated antibodies were incubated for 1 h at room temperature. The blots were developed using a chemioluminescent HRP substrate (Millipore, Billerica, USA). An anti-his antibody (monoclonal #8722 Sigma) was used at 1/2000 dilution for immunoblotting. The anti-Spike RBD mouse monoclonal MAB105420 (R&D systems, Northest Minneapolis, MN, USA) was used at the concentration of 2 μg/mL for immunoblotting.

HEK 293T cells expressing the Spike protein on the cell surface were incubated for 40 min in 3% BSA in PBS buffer and then stained with the mSIP-3022 (1 μg/mL) or MAB105420 (1 μg/mL) antibodies in the same buffer. After washes and incubation with secondary goat anti mouse IgG antibody diluted 1/1000 (Alexa 488 Jackson Immunoflourescence, Cambridge, UK), the cells were analysed with FacsCalibur and Cell-Quest software (Beckton Dickson, Sanjose, CA, USA).

### 2.5. Viral RNA Quantification 

The 812bp SARS-CoV-2_multitarget (MTG) synthetic RNA sequence was synthesized and cloned into the pEX-A128 vector (Eurofins Genomics, Ebersberg, Germany). The primers and probes (FAM-BBQ/BHQ1) for rRT-qPCR are listed in Supplementary Table S1 and include also a specific set for the unique detection of the SARS-CoV-2_MTG as well as of the endogenous RNAse P.

The DNA of pEX-A128-SARS-CoV-2_MTG was linearized with EcoRI and purified by the NucleoSpin Gel and PCR Clean-up kit (Macherey-Nagel catalog no: REF 740609.50). Synthetic RNA was obtained by T7 in vitro transcription with MEGAscript T7 Kit n. AM1333 (ThermoFisher, Rockford, IL, USA) according to manufacturer’s specifications. The RNA was then quantified by UV light absorbance using a nanodrop and loaded on a 6% Acrylamide/8 M Urea gel.

Synthetic and viral RNAs were reverse transcribed and amplified with the Luna Universal Probe One-Step Reaction Mix (New England BioLabs, Ipswich, MA, USA, catalogue no. E3006L). Uniform reaction conditions were set to a 10 min RT reaction at 55 °C, with denaturation at 95 °C for 3 min, 45 cycles of amplification with extension at 58 °C for 30 s, and denaturation at 95 °C for 15 s. Primers were used at the concentrations shown in Supplementary Table S1. The amplification reactions were performed on a BioRad Cfx96 Thermocycler.

More detailed information on the SARS-CoV-2_multitarget (MTG) synthetic RNA is available at https://www.icgeb.org/covid19-resources/ (accessed on 29 April 2021).

### 2.6. High Content Assay

The assay is described in detail elsewhere [30]; briefly, Huh 7-hACE2 cells were seeded overnight to adhere to a 96-well plate, treated with serial dilution of the compounds, and then infected with SARS-CoV-2 with appropriate controls (vehicle, not infected, infected and not treated, and infected and treated with the control inhibitor hydroxychloroquine). The plates were incubated for 20 h at 37 °C, and then fixed with 4% PFA, permeabilized with 0.1% of Triton-X for 15 min and incubated in blocking buffer (PBS containing 1% of BSA). 

The antibody mSIP-3022 was diluted in blocking buffer and incubated for 2 h at 37 °C. The cells were washed twice in PBS and incubated with the secondary antibody AlexaFluor488-conjugated goat anti-mouse IgG (Cat No. A-11001, ThermoFisher, Rockford, IL, USA) plus DAPI for 1 h at 37 °C. Each plate was kept in PBS after washing. Digital images were acquired using the Operetta high content imaging system (Perkin Elmer, Walthem, MA, USA). The digital images were taken from nine different fields of each well. The total number of cells (nuclei) and the number of infected cells were analysed using the Columbus Image Data Storage and Analysis System (Perkin Elmer, Waltham, MA, USA).

### 2.7. Statistics

Typically, three independent experiments in triplicate repeats were conducted for each condition examined. The average values are shown with the standard deviation and *p*-values, measured with a paired two-tailed *t*-test. Only significant *p*-values are indicated by the asterisks above the graphs (** *p* < 0.01 highly significant; * *p* < 0.05 significant). Where asterisks are missing, the differences were calculated to be non-significant (n.s.).

## 3. Results

### 3.1. Anti-SARS-CoV-2 Activity of Miglustat in Vero E6 and Huh7 Cells

**Figure 1:**
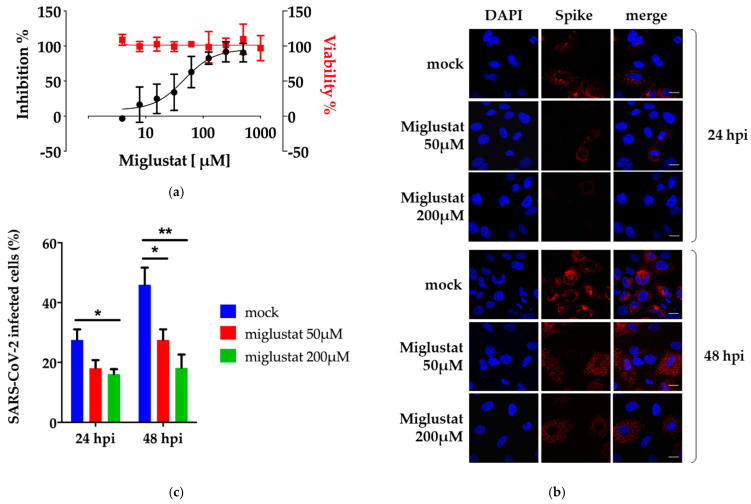
Anti-SARS-CoV-2 activity of Miglustat. (**a**) Antiviral plaque assay. Miglustat at the indicated concentrations was added to Vero E6 monolayers infected with SARS-CoV-2. Following incubation for three days, the cells were fixed and stained to count the viral plaques against vehicle control, which were plotted as the percent inhibitory activity (black dots). The cytotoxicity was measured by the Alamar blue method and data plotted as percent viability (red squares). (**b**) Immunofluorescence assay. Huh7 cells were infected with SARS-CoV-2 moi = 0.1 and incubated with Miglustat as indicated. The cells were then fixed and stained with mSIP-3022 antibody against Spike (red) to acquire confocal images. The nuclei were stained by DAPI. The bar corresponds to 20 μm. (**c**) Quantification of infected cells. SARS-CoV-2 infected cells were counted. The results from 200 cells per condition were plotted as the percent of infected cells. Each pair of mock/treatment conditions was analysed and only significant differences are marked by the asterisks, significant *p*-values are indicated by ** *p* < 0.01 highly significant; * *p* < 0.05 significant, measured with a paired two-tailed *t*-test.

The antiviral properties of Miglustat were initially assessed by performing a plaque assay. SARS-CoV-2 strain ICGEB-FVG_5 was used to infect Vero E6 cells for 1 h. After removal of the inoculum and a wash in PBS, the cells were overlaid with medium containing 1.5% CMC and dilutions of the drug as indicated in Figure 1a. At 72 h post-infection, the cells were fixed and stained to reveal plaques, which were visually counted. In parallel, the cytotoxicity was assessed by the Alamar blue method at the indicated dilutions of drug. The effective concentration for 50% inhibition (EC_50_) of Miglustat was 41 ± 22 μM with no apparent cytotoxicity up to 1000 μM (CC_50_ > 1000 μM). The plaque assay was performed in Vero E6 cells, which is standard for the growth of SARS-CoV-2. However, further analysis would be better performed in cells of human origin.

To establish an infectious model in human cells, a number of available cell lines were tested, including U2OS (osteosarcoma), A549 (adenocarcinoma of the human alveolar basal epithelial), HEK 293 (human embryonic kidney cells), and Huh7 (hepatocellular carcinoma). None of the cell lines tested supported SARS-CoV-2 infection except for the Huh7 hepatocellular carcinoma cell line. As shown in Supplementary Figure S1A, Huh7 supported SARS-CoV-2 infection, albeit delayed by 24 h and at a lower efficiency compared to Vero E6. Miglustat showed an EC_50_ of 13.45 ± 0.7 μM and a CC_50_ > 1000 μM in Huh7 as measured with the virus yield inhibition assay and by the Alamar blue method for cytotoxicity. 

To stain infected cells, a protocol for immunofluorescence was established. To this end, a recombinant monoclonal based on an SIP mouse scaffold (mSIP-3022) was generated carrying the CDR regions of the antibody CR3022 reactive against the receptor binding domain (RBD) of the Spike protein of SARS-CoV-1, which showed high binding affinity also for SARS-CoV-2 Spike protein [27]. As shown in Supplementary Figure S1B, mSIP-3022 efficiently stains the Spike protein in the cytoplasm of infected Vero E6 and Huh7 cells 24 h post-infection.

Next, the efficiency of the SARS-CoV-2 infection of Huh7 cells was assessed in the presence of Miglustat. As shown in Figure 1b and quantified in Figure 1c, Miglustat maintained the number of Huh7 infected cells at the level observed 24 hpi, while the mock treated cells showed an increase of infected cells at 48 hpi as expected from an expansion of the infection in the cell culture. We also noted that the mean fluorescence intensity of the Spike signal decreased significantly in treated cells (Supplementary Figure S2).

Finally, we also measured the inhibitory activity of Miglustat in the human lung cell line Calu-3 to obtain a more physiological cellular model of infection. Miglustat had an EC_50_ of 80.5 ± 23 μM and a CC_50_ > 1000 μM in Calu-3 cells as measured with the virus yield inhibition assay and by the Alamar blue method, respectively.

These data confirm the inhibitory effect of Miglustat in cells of human origin and suggest that the activity of Miglustat is at the level of replication and/or secretion of new infectious virus and not at the binding and entry level.

### 3.2. Dissection of Miglustat Antiviral Activity by Time of Addition Experiments

To better characterize this hypothesis, a time-of-addition (TOA) experiment was performed. Different conditions were used: pre-treatment, co-treatment, and post-treatment. Huh7 cells were pre-treated with 200 μM Miglustat for 3 h and then infected for 1 h in the absence of drug (moi = 0.1). Afterward, the virus was removed and the cells were cultured in drug-free medium until the end of the experiment. For co-treatment, the drug was added together with the virus during infection, and then the cells were maintained in drug-free medium. 

**Figure 2:**
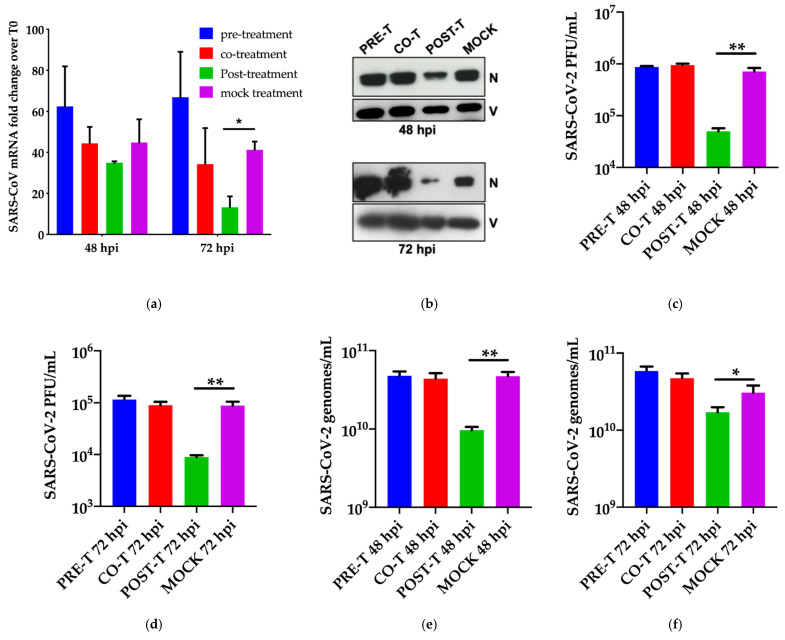

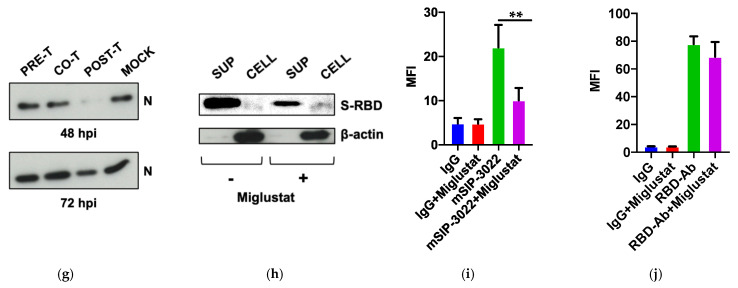
Time of addition studies and the role of Spike. (**a**) Time-of-addition experiment: SARS-CoV-2 genomic RNA. Huh7 cells were infected at moi = 0.1 and incubated with Miglustat before infection (pre-treatment), during infection (co-treatment), and after infection (post-treatment) as described in the text. At the indicated time points, the total RNA was extracted from the infected cells and analysed by RT qPCR. The data are shown as fold-change normalized to their respective T0 values. (**b**) Time-of-addition experiment: SARS-CoV-2 proteins. Protein extracts from Huh7 cells treated as in (**a**) were immunoblotted with a COVID-19 convalescent human serum. The N protein is indicated with Vimentin as the loading control. (**c**,**d**) Time-of-addition experiment: SARS-CoV-2 infectious virus. The infectious virus produced in the experiment was measured as PFU/mL on Vero E6 cells as indicated. (**e**,**f**) Time-of-addition experiment: SARS-CoV-2 secreted genomes. The SARS-CoV-2 genomes in the supernatant of infected cells were quantified by RT qPCR as indicated. (**g**) Time-of-addition experiment: SARS-CoV-2 secreted virions. The virion protein N of secreted SARS-CoV-2 was detected with a convalescent human serum. The loading controls are shown in Figure 2b showing the cell extract results of infected cells. (**h**) Secretion of SARS-CoV-2 Spike RBD. The his-tagged Spike-RBD was expressed for 24 h in HEK-293T cells in the presence of Miglustat and protein detected by immunoblot for the his-tag both in supernatant and cell extracts with β-actin as the loading control. (**i**,**j**) Surface expression of SARS-CoV-2 Spike. Full-length SARS-CoV-2 Spike was expressed in HEK-293T cells for 24 h in the presence of Miglustat and its expression and correct folding on the cell surface was detected with the mSIP-3022 antibody (**i**) or with the anti RBD antibody MAB10540 (**j**). The control was incubated with the secondary antibody only (IgG). Significant *p*-values are indicated by ** *p* < 0.01 highly significant; * *p* < 0.05 significant, measured with a paired two-tailed *t*-test.

For the post-entry experiment, the drug was added at 3 h post-infection and maintained until the end of the experiment. As shown in Figure 2a, addition of the drug did not affect the viral entry, and the drug was not virucidal when administered concomitant with infection. Replication (intracellular viral RNA) was slightly affected at 48 hpi and significantly at 72 hpi consistent with the idea that Miglustat was effective at the post-entry level. This was reflected by the reduction of intracellular nucleocapsid N protein observed at both time points (Figure 2b). 

Interestingly, a strong reduction of infectious virus was observed in the post-entry conditions (Figure 2c,d) paralleled by a decrease of extracellular viral genomes (Figure 2e,f) and N protein (Figure 2g). To note, the quantification of viral genomes was obtained by a method developed for the purpose that takes advantage of a synthetic RNA carrying several of the targets for amplification currently in use (Supplementary Figure S3 and Table S1). This approach is freely available by accessing the ICGEB COVID-19 Resources pages (https://www.icgeb.org/covid19-resources/) (accessed on 29 April 2021).

As we measured only a minor effect of the drug on intracellular viral RNA together with a more pronounced inhibition on released viral genomes and on infectivity, we decided to explore the later stages of the viral lifecycle in greater detail.

### 3.3. Effect of Miglustat on the Spike Protein

We expected Miglustat activity at the post-entry level to target the proper folding of glycoproteins. The Spike protein and its receptor-binding domain are heavily glycosylated and undergo folding and glycosylation through the ER before being secreted and exposed on the plasma membrane. Previous data on the Spike protein of SARS-CoV-1 indicated that both glycosylation and secretion were affected by Miglustat [31,32]. Therefore, we took advantage of an expression vector for SARS-CoV-2 Spike RBD to assess the effect of Miglustat treatment on protein release from transfected 293T cells. As shown in Figure 2h, the protein was highly abundant in the cell supernatant in normal conditions; however, upon treatment with 200 μM Miglustat, the amount of protein in the supernatant was reduced. 

Conversely, RBD was more abundant in the intracellular extracts of treated cells, consistent with the accumulation of misfolded proteins in the ER (Supplementary Figure S4). These data point to the role of Miglustat as an inhibitor of the proper folding and/or release of functional Spike protein. To reinforce this observation, full-length Spike protein was transfected in 293T cells, and the fully folded protein expressed on the cell surface was detected by the conformation-dependent mSIP-3022 antibody (Figure 2i). The antibody CR3022 was not developed for this study as it was discovered against SARS-CoV in 2003 [26] and further characterized for SARS-CoV-2 [27]. 

In this work, we cloned the complementarity-determining region (CDR) in a mouse small immune protein (mSIP) scaffold and verified that it was able to detect Spike in immunofluorescence and flow-cytometry. CR3022 was recently described to bind to Spike RBD of SARS-CoV-2 in a conformation-dependent mode through what is called protein “breathing”, which requires the unmasking of a cryptic epitope bound by the antibody [33]. Indeed, when we repeated the experiment with a different SARS-CoV-2 Spike RBD antibody able to bind a linear epitope (MAB10540), we demonstrated that Spike was still present on the surface of cells expressing Spike and only marginally affected by Miglustat treatment (Figure 2j). These data are not conclusive on the role of Miglustat in Spike folding but are suggestive that the impact of the drug treatment could be both at the secretion and folding level.

### 3.4. Anti-SARS-CoV-2 Activity of Miglustat and Celgosivir

**Figure 3:**
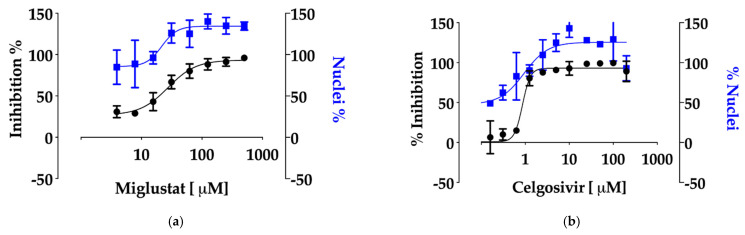
Anti-SARS-CoV-2 activity of Miglustat and Celgosivir. (**a**) Dose response of Miglustat in Huh-7hACE2 cells. Miglustat, at the indicated concentrations, was added to cell monolayers infected with SARS-CoV-2. Following incubation for 20 h, the cells were fixed and stained with the anti-Spike recombinant antibody mSIP-3022. Immunofluorescence images of infected cells (black) and the number of nuclei (blue) were collected and analysed by the Operetta high content imaging system using the Columbus Image Data Storage and Analysis software. (**b**) The dose response of Celgosivir in Huh7-hACE2 cells. Celgosivir, at the indicated concentrations, was added to cell monolayers infected with SARS-CoV-2 and processed as above.

To highlight the role of inhibitors of glycosylases in the context of SARS-CoV-2 infection, both Miglustat and the Castanospermine pro-drug Celgosivir were tested in parallel in a high-throughput assay based on Huh7-hACE2 cells recently developed [30]. As shown in Figure 3a, Miglustat confirmed its antiviral potential with an EC_50_ of 19.9 ± 3.4 μM. Celgosivir was also inhibitory as hypothesized, with a remarkable EC_50_ of 1 ± 0.2 μM (Figure 3b). In both cases, the number of viable nuclei increased upon treatment, a result of the protection from infection and lack of cytotoxicity up to the highest concentrations tested (500 and 200 μM, respectively). CC_50_ was also measured by the Alamar blue assay with values exceeding 1000 μM for both drugs.

## 4. Discussion

Host directed antiviral therapy is a strategy of inhibiting virus infection by targeting host factors that are essential for viral replication [34]. Currently, there is a pressing need for antiviral drugs in the context of SARS-CoV-2 infection. Miglustat is a drug that is in current clinical use for the treatment of certain genetic disorders and was shown to be active against a variety of viral infections making it a suitable candidate for drug repurposing toward SARS-CoV-2 [35]. In this work, the activity of Miglustat against SARS-CoV-2 has been demonstrated in vitro on Vero E6 cells with EC_50_ values ranging from 13 to 80 μM in different cell lines. 

The standard dosage for lysosomal storage diseases, such as Gaucher or Niemann- Pick, is 100 mg/three times a day, with a maximum daily dose of 600 mg/day. A single dose of 100 mg Miglustat reached a peak in plasma concentration of around 3–5 μM within 4 h, while the half-life was approximately 8 h. When this dose was administered every 4 h/six times per day, the plasma concentration of Miglustat stabilized around 10 μM [36]. When 200 mg Miglustat was administered every 8 h/three times a day, the plasma concentration could be also higher than 10 μM in 24 h. However, increased dosage could lead to well-described adverse reactions that include tremors, diarrhoea, numbness, and thrombocytopenia. The concentration of Miglustat at the site of SARS-CoV-2 replication in the lungs is not known.

Miglustat has been shown to act through two different mechanisms: at the level of virus entry, by perturbing the plasma membrane, and at the level of folding and secretion of virion proteins by affecting the essential glycosylation steps in the ER. The first mechanism is not supported by the data since pre-treatment of cells with Miglustat three hours before infection did not inhibit SARS-CoV-2. However, the possibility remains open that Miglustat affects a receptor that has a slow turnover and is affected only marginally in three hours. 

For example, hACE2, the human receptor of Spike, has been shown to be targeted by Miglustat, although the kinetics have not been investigated [37]. The correct folding and secretion of glycoproteins is a process that is tightly controlled in the ER by chaperons, such as Calnexin, that recognize specific glycosylation intermediates [38]. Miglustat interferes with this process resulting in the accumulation of misfolded proteins and a defect in secretion. Consistently, the Spike protein of SARS-CoV-1 was shown to bind Calnexin, and disruption of this function caused a decrease of virus infectivity [32].

The inhibition of glycosylation of viral proteins in the ER exerts a potent antiviral effect, which is further demonstrated by the activity of Celgosivir against SARS-CoV-2, with an EC50 of 1 ± 0.2 μM. Pharmacokinetics data from the CELANDEN clinical trial showed that Celgosivir was rapidly converted to Catastanospermine in vivo with a maximum peak concentration of 30.2 μM and a minimum concentration always above 2 μM throughout the dosing period [21].

After this work was posted as preprint in May 2020, two other reports explored the use of glucosidase inhibitors for the treatment of SARS-CoV-2. Nunez-Santos et al. tested and confirmed Miglustat and Castanospermine, the active component of the prodrug Celgosivir, as glycosylation inhibitors of SARS-CoV-2 Spike and the hACE2 receptor [37]. In their work, they failed to demonstrate a functional effect on syncytia formation or on the interaction of Spike with hACE2. However, their approach was mostly based on transfected Spike and did not consider infection assays. 

ACE2 glycosylation inhibitors have been already shown to be functional in inhibiting other human coronaviruses, thus, expanding the potential of Miglustat for another important target for SARS-CoV-2 infection [39]. The mechanism was not clear, as it appears to have occurred at a step following binding to the receptor. The work of Clarke et al. assessed the activity of a derivative of Miglustat, UV-4, and of Celgosivir on SARS-CoV-2-infected cells and demonstrated inhibitory activity in the micromolar range, in agreement with our work [40]. These different approaches concur in demonstrating that inhibitors of glucosidases are active in infectious assays and in in vitro assays probing Spike and ACE2 proteins. How the inhibitors are actually inhibiting the virus appears to be unrelated to binding to the receptor and, instead, to a post-binding effect.

In conclusion, this work provides in vitro evidence for the repurposing of Miglustat and for the investigational drug Celgosivir as inhibitors of SARS-CoV-2. Consideration of both drugs for clinical trials for the treatment of COVID-19 patients should carefully consider dosing, adverse side effects, and, most importantly, the initiation of treatment with respect to the progress of the disease. Alternative routes of administration, such as by aerosol, could also be envisaged for the treatment of COVID-19 patients. Inhibitors of glycosidases represent a promising class of broad-range antivirals that could represent a first line option for emerging viral infections. 

## Data Availability

Data are available on request from the corresponding author.

## Supplementary Materials

The following are available online at https://www.mdpi.com/article/10.3390/v13050808/s1, Figure S1: (A) SARS-CoV-2 infectivity, (B) Immunofluorescence assay. Figure S2: Effect of Miglustat on the mean fluorescence intensity of Spike staining in Huh7 cells. Figure S3: (A) Sequence of the SARS-CoV-2 MTG synthetic RNA, (B) Quality check of SARS-CoV-2 MTG, (C) Definition of the linear range of amplification for the different primer sets used. Figure S4: Secretion of SARS-CoV-2 Spike RBD. Table S1: Primers and probes used for RT-qPCR.

## References

[1] Zhou P., Yang X.L., Wang X.G., Hu B., Zhang L., Zhang W., Si H.R., Zhu Y., Li B., Huang C.L. (2020). A pneumonia outbreak associated with a new coronavirus of probable bat origin. Nature.

[2] Wu F., Zhao S., Yu B., Chen Y.M., Wang W., Song Z.G., Hu Y., Tao Z.W., Tian J.H., Pei Y.Y. (2020). A new coronavirus associated with human respiratory disease in China. Nature.

[3] WHO World Health Organization-Coronavirus Disease 2019 (COVID-19). Weekly Epidemiological Update. https://www.who.int/publications/m/item/weekly-epidemiological-update-on-covid-19---23-march-2021.

[4] V’Kovski P., Kratzel A., Steiner S., Stalder H., Thiel V. (2020). Coronavirus biology and replication: Implications for SARS-CoV-2. Nat. Rev. Microbiol..

[5] Xie M., Chen Q. (2020). Insight into 2019 novel coronavirus-An updated interim review and lessons from SARS-CoV and MERS-CoV. Int. J. Infect. Dis..

[6] Tay M.Z., Poh C.M., Renia L., MacAry P.A., Ng L.F.P. (2020). The trinity of COVID-19: Immunity, inflammation and intervention. Nat. Rev. Immunol..

[7] Sanders J.M., Monogue M.L., Jodlowski T.Z., Cutrell J.B. (2020). Pharmacologic Treatments for Coronavirus Disease 2019 (COVID-19): A Review. JAMA.

[8] Liu Z., VanBlargan L.A., Bloyet L.M., Rothlauf P.W., Chen R.E., Stumpf S., Zhao H., Errico J.M., Theel E.S., Liebeskind M.J. (2021). Identification of SARS-CoV-2 spike mutations that attenuate monoclonal and serum antibody neutralization. Cell Host. Microbe.

[9] Elbein A.D. (1991). Glycosidase inhibitors: Inhibitors of N-linked oligosaccharide processing. FASEB J..

[10] Fischl M.A., Resnick L., Coombs R., Kremer A.B., Pottage J.C., Fass R.J., Fife K.H., Powderly W.G., Collier A.C., Aspinall R.L. (1994). The safety and efficacy of combination N-butyl-deoxynojirimycin (SC-48334) and zidovudine in patients with HIV-1 infection and 200-500 CD4 cells/mm3. J. Acquir. Immune Defic. Syndr..

[11] Tierney M., Pottage J., Kessler H., Fischl M., Richman D., Merigan T., Powderly W., Smith S., Karim A., Sherman J. (1995). The tolerability and pharmacokinetics of N-butyl-deoxynojirimycin in patients with advanced HIV disease (ACTG 100). The AIDS Clinical Trials Group (ACTG) of the National Institute of Allergy and Infectious Diseases. J. Acquir. Immune Defic. Syndr. Hum. Retrovirol..

[12] Dwek R.A., Butters T.D., Platt F.M., Zitzmann N. (2002). Targeting glycosylation as a therapeutic approach. Nat. Rev. Drug Discov.

[13] Chang J., Warren T.K., Zhao X., Gill T., Guo F., Wang L., Comunale M.A., Du Y., Alonzi D.S., Yu W. (2013). Small molecule inhibitors of ER alpha-glucosidases are active against multiple hemorrhagic fever viruses. Antiviral. Res..

[14] Wu S.F., Lee C.J., Liao C.L., Dwek R.A., Zitzmann N., Lin Y.L. (2002). Antiviral effects of an iminosugar derivative on flavivirus infections. J. Virol..

[15] Platt F.M., d’Azzo A., Davidson B.L., Neufeld E.F., Tifft C.J. (2018). Lysosomal storage diseases. Nat. Rev. Dis. Primers.

[16] Sunkara P.S., Taylor D.L., Kang M.S., Bowlin T.L., Liu P.S., Tyms A.S., Sjoerdsma A. (1989). Anti-HIV activity of castanospermine analogues. Lancet.

[17] Taylor D.L., Sunkara P.S., Liu P.S., Kang M.S., Bowlin T.L., Tyms A.S. (1991). 6-0-butanoylcastanospermine (MDL 28,574) inhibits glycoprotein processing and the growth of HIVs. AIDS.

[18] Durantel D. (2009). Celgosivir, an alpha-glucosidase I inhibitor for the potential treatment of HCV infection. Curr. Opin. Investig. Drugs.

[19] Low J.G., Sung C., Wijaya L., Wei Y., Rathore A.P.S., Watanabe S., Tan B.H., Toh L., Chua L.T., Hou Y. (2014). Efficacy and safety of celgosivir in patients with dengue fever (CELADEN): A phase 1b, randomised, double-blind, placebo-controlled, proof-of-concept trial. Lancet Infect. Dis..

[20] Watanabe S., Chan K.W., Dow G., Ooi E.E., Low J.G., Vasudevan S.G. (2016). Optimizing celgosivir therapy in mouse models of dengue virus infection of serotypes 1 and 2: The search for a window for potential therapeutic efficacy. Antiviral. Res..

[21] Sung C., Wei Y., Watanabe S., Lee H.S., Khoo Y.M., Fan L., Rathore A.P., Chan K.W., Choy M.M., Kamaraj U.S. (2016). Extended Evaluation of Virological, Immunological and Pharmacokinetic Endpoints of CELADEN: A Randomized, Placebo-Controlled Trial of Celgosivir in Dengue Fever Patients. PLoS Negl. Trop. Dis..

[22] Milani M., Donalisio M., Milan Bonotto R., Scheneider E., Arduino I., Boni F., Lembo D., Marcello A., Mastrangelo E. (2020). Combined in silico docking and in vitro antiviral testing for drug repurposing identified lurasidone and elbasvir as SARS-CoV-2 and HCoV-OC43 inhibitors. BioRxiv.

[23] Licastro D., Rajasekharan S., Dal Monego S., Segat L., D’Agaro P., Marcello A. (2020). Isolation and Full-Length Genome Characterization of SARS-CoV-2 from COVID-19 Cases in Northern Italy. J. Virol..

[24] Stadlbauer D., Amanat F., Chromikova V., Jiang K., Strohmeier S., Arunkumar G.A., Tan J., Bhavsar D., Capuano C., Kirkpatrick E. (2020). SARS-CoV-2 Seroconversion in Humans: A Detailed Protocol for a Serological Assay, Antigen Production, and Test Setup. Curr. Protoc. Microbiol..

[25] Marcello A., Civra A., Milan Bonotto R., Nascimento Alves L., Rajasekharan S., Giacobone C., Caccia C., Cavalli R., Adami M., Brambilla P. (2020). The cholesterol metabolite 27-hydroxycholesterol inhibits SARS-CoV-2 and is markedly decreased in COVID-19 patients. Redox. Biol..

[26] ter Meulen J., van den Brink E.N., Poon L.L., Marissen W.E., Leung C.S., Cox F., Cheung C.Y., Bakker A.Q., Bogaards J.A., van Deventer E. (2006). Human monoclonal antibody combination against SARS coronavirus: Synergy and coverage of escape mutants. PLoS Med..

[27] Tian X., Li C., Huang A., Xia S., Lu S., Shi Z., Lu L., Jiang S., Yang Z., Wu Y. (2020). Potent binding of 2019 novel coronavirus spike protein by a SARS coronavirus-specific human monoclonal antibody. Emerg. Microbes Infect..

[28] Petris G., Bestagno M., Arnoldi F., Burrone O.R. (2014). New tags for recombinant protein detection and O-glycosylation reporters. PLoS ONE.

[29] Carletti T., Zakaria M.K., Faoro V., Reale L., Kazungu Y., Licastro D., Marcello A. (2019). Viral priming of cell intrinsic innate antiviral signaling by the unfolded protein response. Nat. Commun..

[30] Milani M., Donalisio M., Bonotto R.M., Schneider E., Arduino I., Boni F., Lembo D., Marcello A., Mastrangelo E. (2021). Combined in silico and in vitro approaches identified the antipsychotic drug lurasidone and the antiviral drug elbasvir as SARS-CoV2 and HCoV-OC43 inhibitors. Antiviral. Res..

[31] Ritchie G., Harvey D.J., Feldmann F., Stroeher U., Feldmann H., Royle L., Dwek R.A., Rudd P.M. (2010). Identification of N-linked carbohydrates from severe acute respiratory syndrome (SARS) spike glycoprotein. Virology.

[32] Fukushi M., Yoshinaka Y., Matsuoka Y., Hatakeyama S., Ishizaka Y., Kirikae T., Sasazuki T., Miyoshi-Akiyama T. (2012). Monitoring of S protein maturation in the endoplasmic reticulum by calnexin is important for the infectivity of severe acute respiratory syndrome coronavirus. J. Virol..

[33] Yuan M., Wu N.C., Zhu X., Lee C.D., So R.T.Y., Lv H., Mok C.K.P., Wilson I.A. (2020). A highly conserved cryptic epitope in the receptor binding domains of SARS-CoV-2 and SARS-CoV. Science.

[34] Zakaria M.K., Carletti T., Marcello A. (2018). Cellular Targets for the Treatment of Flavivirus Infections. Front. Cell Infect. Microbiol..

[35] Williams S.J., Goddard-Borger E.D. (2020). alpha-glucosidase inhibitors as host-directed antiviral agents with potential for the treatment of COVID-19. Biochem Soc. Trans..

[36] van Giersbergen P.L., Dingemanse J. (2007). Influence of food intake on the pharmacokinetics of miglustat, an inhibitor of glucosylceramide synthase. J. Clin. Pharmacol..

[37] Nunes-Santos C.J., Kuehn H.S., Rosenzweig S.D. (2021). N-Glycan Modification in Covid-19 Pathophysiology: In vitro Structural Changes with Limited Functional Effects. J. Clin. Immunol..

[38] Helenius A., Aebi M. (2001). Intracellular functions of N-linked glycans. Science.

[39] Zhao X., Guo F., Comunale M.A., Mehta A., Sehgal M., Jain P., Cuconati A., Lin H., Block T.M., Chang J. (2015). Inhibition of endoplasmic reticulum-resident glucosidases impairs severe acute respiratory syndrome coronavirus and human coronavirus NL63 spike protein-mediated entry by altering the glycan processing of angiotensin I-converting enzyme 2. Antimicrob. Agents Chemother..

[40] Clarke E.C., Nofchissey R.A., Ye C., Bradfute S.B. (2020). The iminosugars celgosivir, castanospermine and UV-4 inhibit SARS-CoV-2 replication. Glycobiology.

