# Supplementary material for: Inhibitors of Protein Glycosylation Are Active against the Coronavirus Severe Acute Respiratory Syndrome Coronavirus SARS-CoV-2
Source: Viruses. 2021 Apr 30;13(5):808. doi: 10.3390/v13050808 (PMC8144969; doi:10.3390/v13050808)
Supplement: Supplementary file 1 [file viruses-13-00808-s001.zip › viruses-1116498-supplementary.pdf]

viruses

MDPI

Supplementary Materials: Inhibitors of Protein Glycosylation are Active against the Coronavirus Severe Acute Respiratory Syndrome Coronavirus SARS-CoV-2

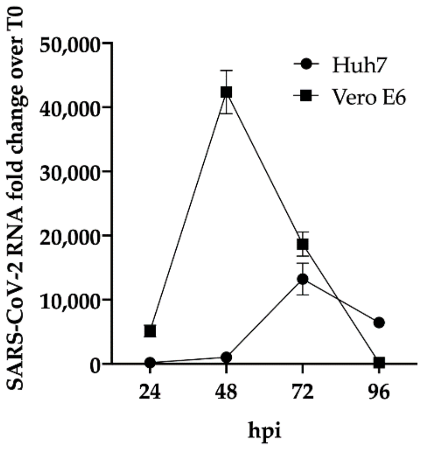

Copyright: © 2021 by the authors. Licensee MDPI, Basel, Switzerland. This article is an open access article distributed under the terms and conditions of the Creative Commons Attribution (CC BY) license (http://creativecommons.org/licenses/by/4.0/).

(a)

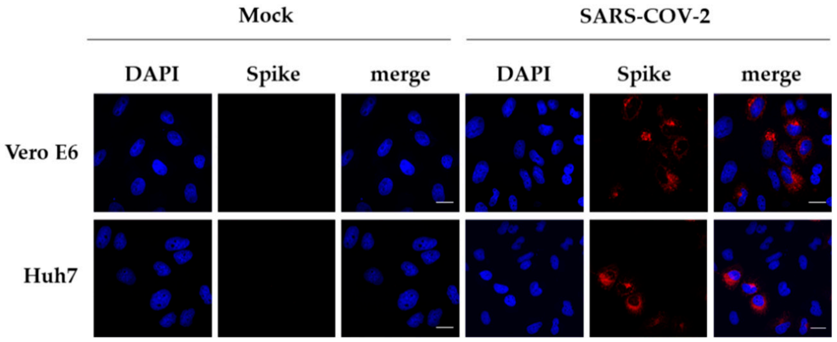

(b)

Figure S1. (**a**) SARS-CoV-2 infectivity. Vero E6 and Huh7 cells were infected in parallel with SARS-CoV-2 at moi 0.1. At the indicated time points RNA was extracted from the cells and measured by RT qPCR for SARS-CoV-2 genomes. Data are plotted as fold change over T0; (**b**) Immunofluorescence assay. Vero E6 and Huh7 cells were infected or mock infected with SARS-CoV-2 moi = 0.1 for 24 h. Cells were then fixed and stained with mSIP-3022 antibody against Spike (red) to acquire confocal images. Nuclei are stained by DAPI. Bar corresponds to 20 µm.

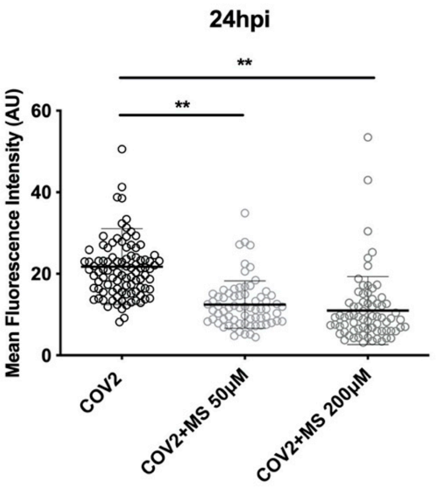

(a)

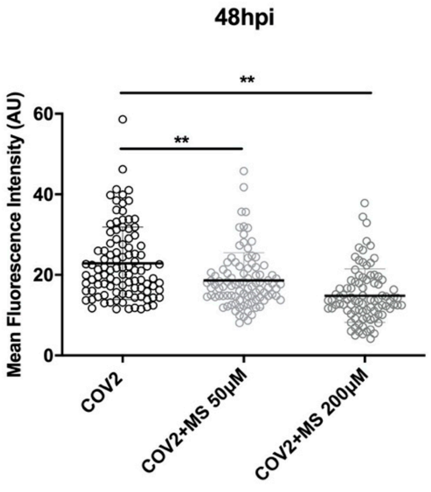

(b)

Figure S2. Effect of Miglustat (MS) on the mean fluorescence intensity of Spike staining in Huh7 cells. Huh7 cells were infected with SARS-CoV-2 moi = 0.1 and incubated with Miglustat for 24 hours (a) or 48 hours (b) as indicated. Cells were

then fixed and stained with mSIP-3022 antibody against Spike (red) to acquire confocal images as described for the experiment shown in Figure 1B and 1C. Mean fluorescence intensity of the Spike signal was quantified by Image J. Results from 200 cells per condition were plotted in arbitrary units.

```
TAATACGACTCACTATAGGGAGAGGGGAACTTCTCCTGCTAGAATGGCTGGCAATGGCGGTGA
TGCTGCTCTTGCTTTGCTGCTGCTTGACAGATTGAACCAGCTTGAGAGCAAAATGTCTGACAG
GTACGTTAATAGTTAATAGCGTACTTCTTTTTCTTGCTTTCGTGGTATTCTTGCTAGTTACAC
TAGCCATCCTTACTGCGCTTCGATTGTGTGCGTACTGCTGCAATATTGGTGAAATGGTCATGT
GTGGCGGTTCACTATATGTTAAACCAGGTGGAACCTCATCAGGAGATGCCACAACTGCTTATG
CTAATAGTGTTTTTAACATTTGGGGAGCCTTGAATACACCAAAAGATCACATTGGCACCCGCA
ATCCTGCTAACAATGCTGCAATCGTGCTACACTTCCTCAAGGAACAACATTGCCAAAAGGCTT
CTACGCAGAAGGGAGCAGAGGCGGCAGTCAAGCCTCTTCTCGTTCCTCGACCCCAAAATCAGC
GAAATGCACCCCGCATTACGTTTGGTGGACCCTCAGATTCAACTGGCAGTAACCAGATTACAA
ACATTGGCCGCAAATTGCACAATTTGCCCCCAGCGCTTCAGCGTTCTTCGGAATGTCGCGCAG
ATTTGGACCTGCGAGCGGGTTCTGACCTGAAGGCTCTGCGCGGACTTGTGGAGACAGCCGCTC
CCCTGTGGGTTTTACACTTAAAAACACAGTCTGTACCGTCTGCGGTATGTGGAAAGGTTATGG
CTGTAGTTGTGATCAACTCCGCGAACCCATGCTTCAGTCAGCTGATGCACAATCGT
```

(a)

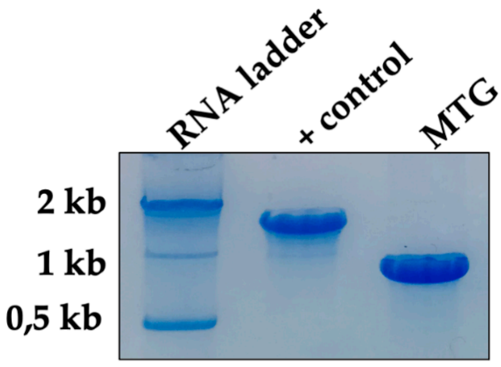

(b)

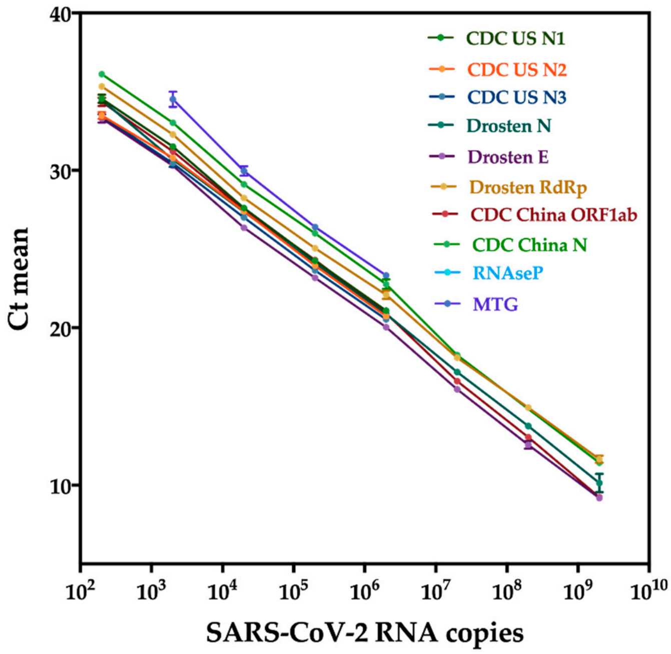

(c)

Figure S3. (**a**) Sequence of the SARS-CoV-2 MTG synthetic RNA. The construct was designed and optimized to carry the following sequences in sequential order: T7, CDC China N, Drosten/Charité E (E-Sarbeco), Drosten/Charité RdRp (RdRp-SARS), Drosten/Charité N (N-Sarbeco), CDC US N3, CDC US N1, CDC US N2, RNAse P and CDC China ORF1ab; (**b**) Quality check of SARS-CoV-2 MTG. RNA (1 µg) was loaded on a 6% Acrylamide/ 8 M Urea gel: lane 1: ssRNA ladder, lane 2: positive control (MegaScript), lane 3: SARS-CoV-2 MTG. RNA; (**c**)Definition of the linear range of amplification for the different primer sets used. Optimal dilutions of SARS-CoV-2 MTG RNA were established for each primer set. Plot shows quantified RNA genomes against Ct values and was used for quantification purposes.

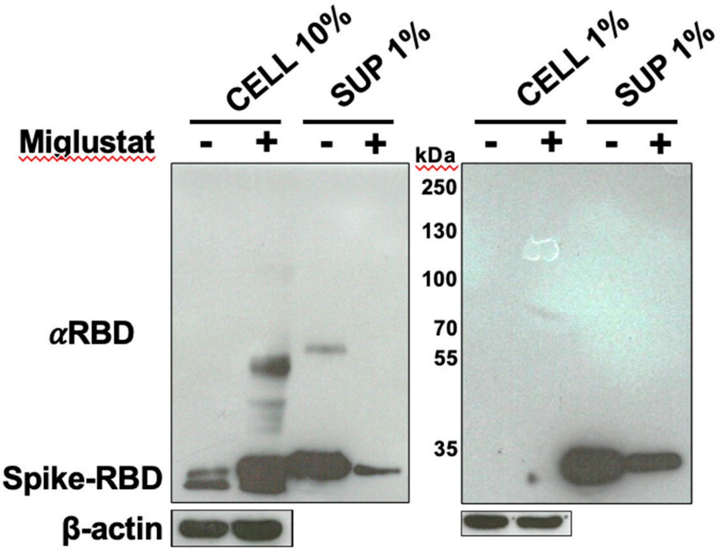

Figure S4. Secretion of SARS-CoV-2 Spike RBD. The his-tagged Spike-RBD was expressed for 24 h in HEK-293T cells in the presence of Miglustat as described in Figure 2H. Immunoblot with MAB10540 against RBD shows the cell associated amount of protein (cell) and the secreted (sup). Due to the different amount of protein in the two compartments a second blot was performed by loading 10× more cell extract (left panel). β-actin is the loading control in cell extracts.

Table S1. Primers and probes used for RT-qPCR.

Oligo Name	Sequence (5′>3′)	Working con.
MTG-specific-Fwd	GGAGAGGGGAACTTCTCCTG	1 µM
MTG-specific-probe	FAM-TGAACCAGCTTGAGAGCAAAATGTCTGACAGGTACG-BHQ1	0.5 µM
MTG-specific-Rev	CACGAAAGCAAGAAAAAGAAGTACGC	1 µM
CDCchinaORF1ab-Fwd	CCCTGTGGGTTTTACACTTAA	1 µM
CDCchinaORF1ab-Probe	FAM-CCGTCTGCGGTATGTGGAAAGGTTATGG-BHQ1	0.5 µM
CDCchinaORF1ab-Rev	ACGATTGTGCATCAGCTGA	1 µM
CDCchinaN-Fwd	GGGGAACTTCTCCTGCTAGAAT	1 µM
CDCchinaN-Probe	FAM-TTGCTGCTGCTTGACAGATT-BHQ1	0.5 µM
CDCchinaN-Rev	CAGACATTTTGCTCTCAAGCTG	1 µM
E-Sarbeco-Fwd	ACAGGTACGTTAATAGTTAATAGCGT	0.4 µM
E-Sarbeco-Probe	FAM-ACACTAGCCATCCTTACTGCGCTTCG-BBQ	0.2 µM
E-Sarbeco-Rev	ATATTGCAGCAGTACGCACACA	0.4 µM
RdRp-SARSr-Fwd	GTGAAATGGTCATGTGTGGCGG	0.6 µM
RdRp-SARSr-Probe2	FAM-CAGGTGGAACCTCATCAGGAGATGC-BBQ	0.2 µM
RdRp-SARSr-Rev	CAAATGTTAAAAACACTATTAGCATA	0.8 µM
N-Sarbeco-Fwd	CACATTGGCACCCGCAATC	0.6 µM
N-Sarbeco-Probe	FAM-ACTTCCTCAAGGAACAACATTGCCA-BBQ	0.2 µM
N-Sarbeco-Rev	GAGGAACGAGAAGAGGCTTG	0.8 µM
2019-nCoV-N1-Fwd	GACCCCAAAATCAGCGAAAT	1 µM
2019-nCoV-N1-Probe	FAM-ACCCCGCATTACGTTTGGTGGACC-BHQ1	0.5 µM
2019-nCoV-N1-Rev	TCTGGTTACTGCCAGTTGAATCTG	1 µM
2019-nCoV-N2-Fwd	TTACAAACATTGGCCGCAAA	1 µM
2019-nCoV-N2-Probe	FAM-ACAATTTGCCCCCAGCGCTTCAG-BHQ1	0.5 µM
2019-nCoV-N2-Rev	GCGCGACATTCCGAAGAA	1 µM
2019-nCoV-N3-Fwd	GGGAGCCTTGAATACACCAAAA	1 µM
2019-nCoV-N3-Probe	FAM-ATCACATTGGCACCCGCAATCCTG-BHQ1	0.5 µM
2019-nCoV-N3-Rev	TGTAGCACGATTGCAGCATTG	1 µM

RP-Fwd	AGATTTGGACCTGCGAGCG	1 μM
RP-Probe	FAM-TTCTGACCTGAAGGCTCTGCGCG-BHQ1	0.5 μM
RP-Rev	GAGCGGCTGTCTCCACAAGT	1 μM
